# Novel *KCNH2* and *SLC4A3* variants in short QT syndrome: Clinical phenotypes and antiarrhythmic drug response

**DOI:** 10.1016/j.hrcr.2026.01.015

**Published:** 2026-01-28

**Authors:** Noriko Fukue, Takeshi Ueyama, Seiko Ohno, Masashi Kanemoto, Koichi Kato, Hideaki Akase, Shinichi Okuda, Fumiaki Nakao, Yasuhiro Ikeda

**Affiliations:** 1 Department of Cardiology, Yamaguchi Prefectural Grand Medical Center, Hofu, Yamaguchi, Japan; 2 Wellness Science Center, Yamaguchi University, Yamaguchi, Japan; 3 Medical Genome Center, National Cerebral and Cardiovascular Center, Suita, Japan; 4 Department of Cardiology, Saiseikai Yamaguchi General Hospital, Yamaguchi, Japan; 5 Department of Cardiovascular Medicine, Shiga University of Medical Science, Otsu, Japan; 6 Department of Clinical Laboratory Sciences, Faculty of Medicine and Health Sciences, Yamaguchi University Graduate School of Medicine, Ube, Yamaguchi, Japan

**Keywords:** Short QT syndrome, *KCNH2*, *SLC4A3*, Sudden cardiac death, Potassium channel blocker

**Key Teaching Points**

• Short QT syndrome shows substantial heterogeneity in clinical presentation, age of onset, and circumstances of sudden cardiac events, which may differ according to the underlying genotype.
• Novel variants in *KCNH2* and *SLC4A3* were associated with distinct electrocardiographic characteristics, including differences in T-wave morphology and T peak–T end/corrected QT interval ratio, suggesting genotype-dependent repolarization abnormalities.
• Antiarrhythmic drugs with potassium channel–blocking effects, including nifekalant, bepridil, and quinidine, were associated with QT interval prolongation toward or into the normal range in patients with both *KCNH2* and *SLC4A3* variants.

## Introduction

Short QT syndrome (SQTS) is an inherited condition characterized by distinct short QT intervals on electrocardiography (ECG) that potentially leads to ventricular fibrillation (VF) and sudden cardiac death (SCD).

We studied 2 families with SQTS harboring novel *KCNH2* and *SLC4A3* variants and both exhibiting a high frequency of sudden death. Although several ECG characteristics and pharmacologic approaches have been proposed for the diagnosis and management of SQTS,^1^, ^2^, ^3^, ^4^, ^5^, ^6^ genotype-phenotype correlations and optimal treatment strategies remain incompletely understood. This study aimed to compare the clinical manifestations and ECG features between the 2 families and describe their responses to antiarrhythmic drugs.

## Methods

### Study population and ECG measurements

This study consisted of 2 Japanese SQTS probands and their family members who consulted at our hospital between 1996 and 2024. They provided a written informed consent in accordance with the principles of the Declaration of Helsinki. The study protocol was approved by the ethics committee of the Yamaguchi Grand Medical Center (approval number: 2023-013).

The SQTS diagnosis was based on the 2022 European Society of Cardiology guidelines.^1^ We focused on the investigation of various ECG parameters previously suggested as potential diagnostic and risk-stratification markers for SQTS. Diagnostic ECG parameters include PQ segment depression (PQD)^3^ and shortened J point–T peak (JTp) intervals,^4^ whereas early repolarization (ER) serves as a parameter for risk stratification.^5^ The T peak–T end (TpTe)/corrected QT interval (QTc) ratio was calculated to determine transmural dispersion of repolarization.^4^^,^^6^ We measured these parameters from the precordial lead that showed the highest T-wave amplitude: intervals of RR, QT, JTp, J point–T end, TpTe, and T-wave amplitude.^5^ The tangential method was used to confirm the end of the T wave. All repolarization interval measurements were corrected for RR intervals using Bazett’s formula. TpTe/QTc ratio corrected was T peak–T end c (TpTec) divided by QTc. ER and PQD were evaluated across all leads.^3^^,^^5^ ER was defined as a J point elevation of ≥0.1 mV manifesting as QRS slurring or notching in more than 2 leads.^5^ PQD was defined as ≥0.05 mV (0.5 mm) depression from the isoelectric line between T-wave end and subsequent P-wave onset.^3^

### Genetic testing

After obtaining informed consent, genomic DNA was extracted from peripheral lymphocytes. Genetic testing was performed for probands using the targeted gene sequencing methods reported previously.^7^ The evaluation of the detected variants was classified based on the American College of Medical Genetics and Genomics (ACMG) criteria.^8^^,^^9^

### Drug challenge tests

To evaluate the effect of antiarrhythmic drugs, we administered nifekalant, a class III agent typically used for ventricular arrhythmias, at a dose of 0.3 mg/kg over 5 minutes, followed by a continuous infusion of 0.4 mg/kg/h for 30 minutes intravenously. For ongoing SQTS therapy, we used various antiarrhythmic agents including nifekalant, bepridil, quinidine, and disopyramide. QT intervals were measured after the administration of these drugs.

### Statistical analysis

Normally distributed variables are expressed as means ± standard deviations (SDs), whereas non-normally distributed variables are presented as medians with interquartile ranges (25th and 75th percentiles). ECG measurements were compared using an unpaired *t* test for normally distributed continuous data and the Mann-Whitney U test for non-normally distributed data using SPSS version 29 (IBM Inc, Armonk, NY). The significance level was set at an alpha level of <0.05.

## Results

### Novel variants in SQTS

We identified a novel *KCNH2* variant (NM_000238.4:c.1855T>G, p.F619V.) in family 1 and 2 novel variants, *KCNH2* (NM_000238.4:c.208C>T, p.H70Y) and *SLC4A3* (NM_201574.3:c.1059C>A, p.N353K), in family 2. All variants were evaluated based on the ACMG criteria.^8^

*KCNH2*-F619V is absent in population databases (PM2); is predicted as deleterious in multiple in silico prediction tools (PP3); is a mutational hot spot (PM1), which was supported by functional analysis of *KCNH2*-T618I^10^; and shows cosegregation with the disease in multiple affected family members (PP1). If we classify PM2 as supporting based on the Sequence Variant Interpretation recommendation published in 2020,^9^ *KCNH2*-F619V is classified as a variant of unknown significance (VUS) with 1 moderate and 3 supporting. If we could obtain the functional analysis data of this variant, the pathogenicity based on the ACMG criteria would be changed. In the previous report,^11^ *KCNH2*-H70Y did not affect the rapid component of the delayed rectifier potassium current, and *SLC4A3*-N353K demonstrated loss of function in the functional analysis. From these data, *SLC4A3*-N353K is classified as a likely pathogenic variant with PM2, PS3, PP3, and PP1. *KCNH2*-H70Y is a VUS, with PM2, BS3, and PP1.

### Clinical course of patients with SQTS in 2 families

Detailed clinical courses of patients with SQTS are presented in Supplemental Table 1.

#### Family 1

**Figure 1:**
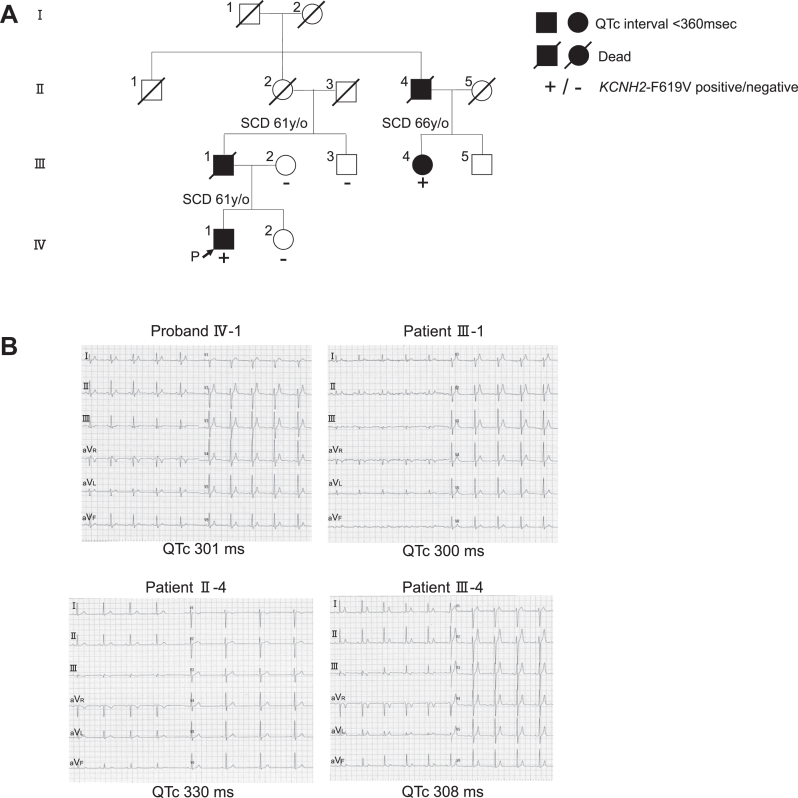
Family 1 with SQTS. **A:** Pedigree of family 1. **B:** ECG recordings of family members with SQTS. ECG = electrocardiography; QTc = corrected QT interval; SCD = sudden cardiac death; SQTS = short QT syndrome; y/o = years old.

The pedigree of the family with the novel *KCNH2* missense variant c.1855T>G, p.F619V, is presented in Figure 1A. The ECG of the SQTS family members is presented in Figure 1B. A 27-year-old man (Ⅳ-1) was noted to have a short QT interval during a medical checkup. Despite a shortened QT interval (QTc 301 ms) on further examination, the patient remained asymptomatic.

**Figure 2:**
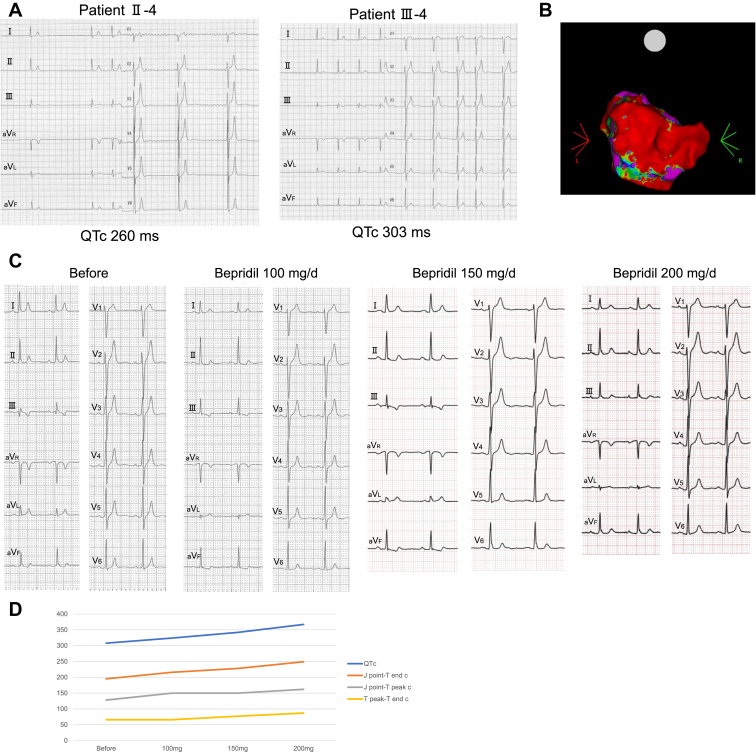
Atrial fibrillation in patients with SQTS carrying the *KCNH2*-F619V. **A:** ECG recordings showing atrial fibrillation in family 1 with SQTS. **B:** Left atrial voltage mapping of patient Ⅲ-4 after left atrial ablation. **C:** ECG recordings after oral bepridil administration of patient Ⅲ-4 from family 1. **D:** Graphs illustrating the dose-dependent prolongation of QTc, J point–T end c, J point–T peak c, and T peak–T end c intervals with bepridil administration (mg/d). ECG = electrocardiography; QTc = corrected QT interval; SQTS = short QT syndrome.

Paroxysmal atrial fibrillation (AF) was noted in his grandmother’s brother (II-4) and his daughter (III-4) (Figure 2A), in addition to a short QT interval (II-4, QTc 330 ms; III-4, QTc 308 ms) (Figure 1B, lower right and left). Although she underwent pulmonary vein isolation along with superior vena cava and left atrial posterior wall isolation (Figure 2B), the daughter experienced recurrent AF. We initiated the administration of bepridil. The ECG after dose modification is presented in Figure 2C. We observed dose-dependent QT interval prolongation. After initiating oral bepridil at 150 mg/d, AF episodes ceased, and the QT interval prolonged (QTc 308–367 ms), reaching the normal range (Figure 2D).

**Figure 3:**
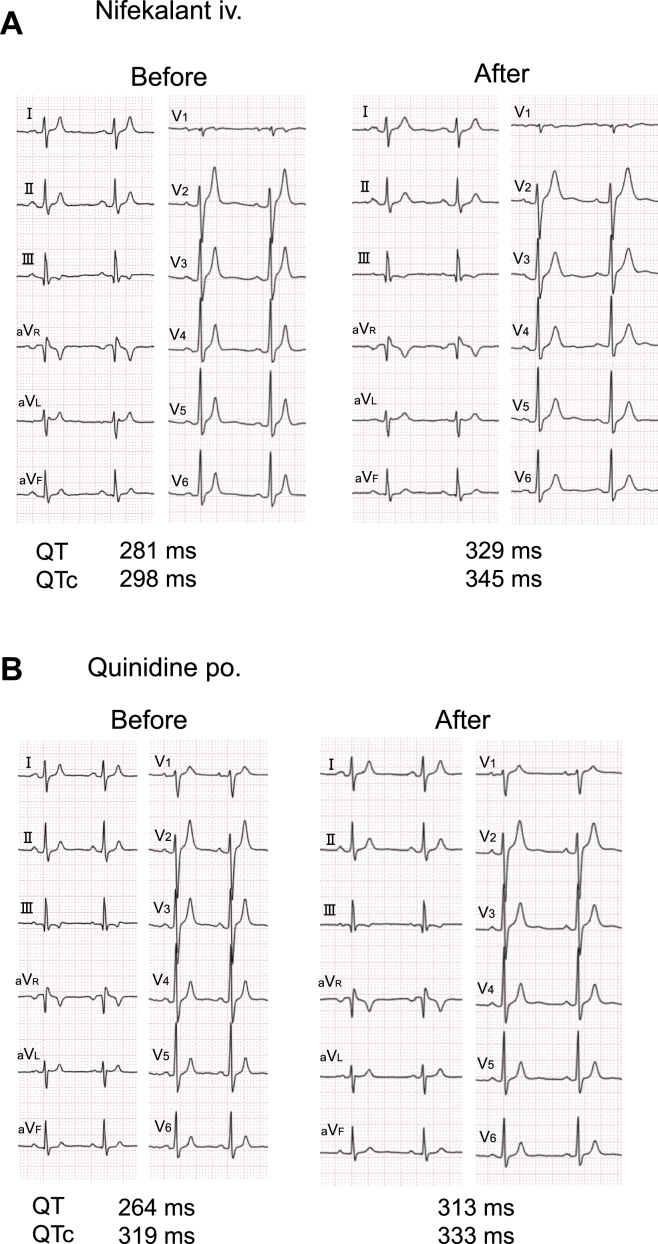
ECG recordings under antiarrhythmic drug administration in proband Ⅳ-1 from family 1. **A:** ECG recordings during nifekalant loading tests. **B:** ECG recordings before and after oral administration of quinidine at 200 mg/d. ECG = electrocardiography; iv. = intravenous; po. = per os; QTc = corrected QT interval.

To evaluate the efficacy of a potassium channel blocker, we administered nifekalant to the proband (Ⅳ-1), which was associated with QT interval prolongation (QTc 298–345 ms) (Figure 3A). We have initiated low-dose quinidine therapy, with plans for gradual dose escalation. Oral quinidine at 200 mg/d resulted in modest QT interval prolongation (Figure 3B).

#### Family 2

**Figure 4:**
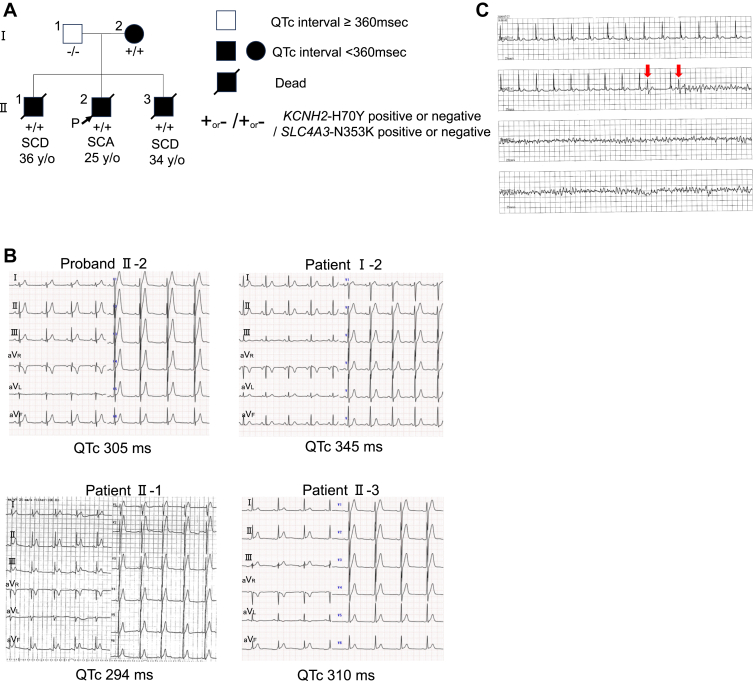
Family 2 with SQTS. **A:** Pedigree of family 2. **B:** ECG recordings of family 2 members with SQTS. **C:** ECG recordings showing ventricular fibrillation of proband Ⅱ-2. *Red arrows* indicate premature ventricular contractions. ECG = electrocardiography; QTc = corrected QT interval; SCA = sudden cardiac arrest; SCD = sudden cardiac death; SQTS = short QT syndrome; y/o = years old.

The second family carried double novel variants: *KCNH2* c.208C>T, p.H70Y, and *SLC4A3* c.1059C>A, p.N353K. The family pedigree is shown in Figure 4A. In 2009, the proband (Ⅱ-2) experienced a cardiac arrest during nighttime sleep at the age of 25 years. Upon arrival at the hospital, VF was detected, and multiple cardioversions were attempted using intravenous amiodarone. However, VF persisted. Finally, cardioversion with intravenous nifekalant successfully terminated the VF. After nifekalant administration, VF did not occur. His ECG recorded at the age of 15 years revealed a short QT interval (QTc 305 ms) (Figure 4B, upper left). ECGs of his mother (Ⅰ-2) and brothers (Ⅱ-1 and Ⅱ-3) also demonstrated short QT intervals (Figure 4B).

**Figure 5:**
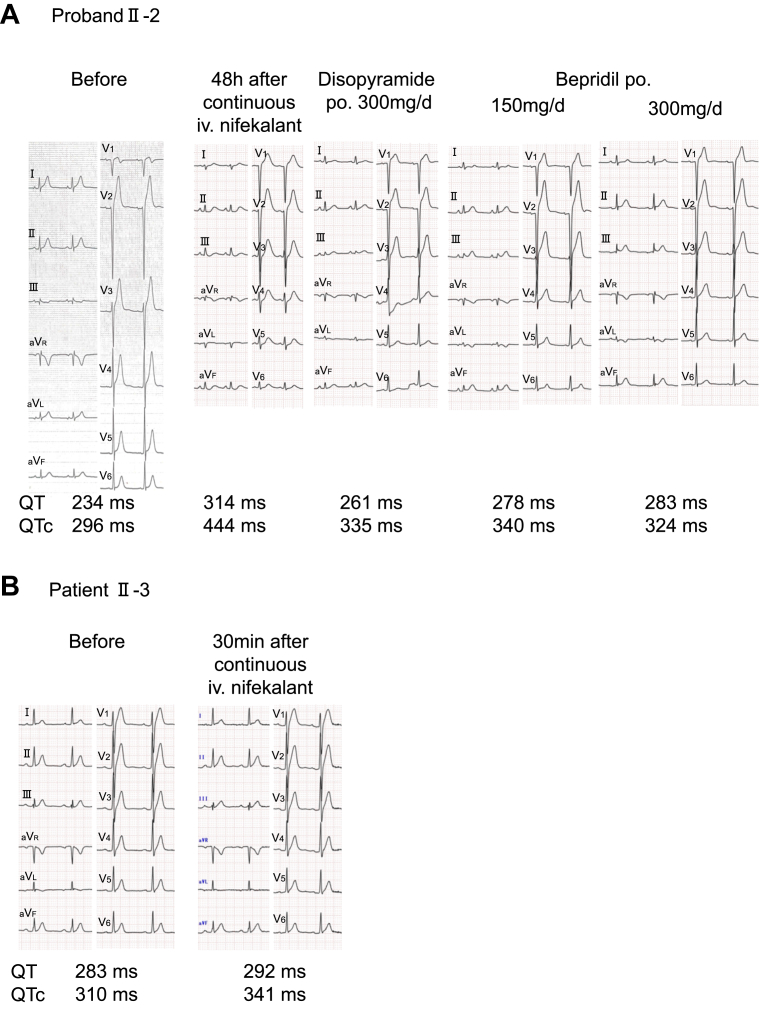
ECG recordings under antiarrhythmic drug administration in family 2. **A:** ECG recordings of treatment with antiarrhythmic drugs for proband Ⅱ-2. **B:** ECG recordings of the nifekalant loading tests for patient Ⅱ-3. ECG = electrocardiography; iv. = intravenous; po. = per os; QTc = corrected QT interval.

At the time, no established guidelines existed for pharmacologic management of SQTS. Multiple potassium channel–blocking agents were administered to the proband (II-2) based on previously published reports.^12^^,^^13^ By the administration of various antiarrhythmic drugs with potassium channel–blocking effect to Ⅱ-2 and II-3, we confirmed the QT interval prolongation in all drugs (Figure 5A and 5B).

### Clinical manifestations in 2 families

Clinical characteristics, ECG measurements, and pharmacologic effects are presented in Supplemental Table 2. The prevalence of SQTS and occurrence of VF and SCD were high in male patients. Cardiac events occurred during work hours in family 1 and nighttime sleep in family 2. In family 2, events occurred in patients aged 25–36 years, whereas, in family 1, events occurred in patients in their 60s.

The ECG measurements of patients with SQTS in 2 families are presented in Supplemental Table 3. The TpTec interval in family 1 was significantly shorter than in family 2 (mean ± SD 69.5 ± 7.5 vs 90.0 ± 5.6 ms; *P* = .005). The TpTe/QTc ratio corrected in family 2 was significantly increased than in family1 (mean ± SD 0.29 ± 0.3 vs 0.23 ± 0.3; *P* = .033). Antiarrhythmic drugs with potassium channel–blocking effects were administered to 2 patients in each family. QT prolongation was observed in both families after administration of nifekalant and bepridil.

## Discussion

### Pathogenicity of variants

We identified a novel variant, *KCNH2*-F619V, in patients with SQTS in family 1. Although it is classified as a VUS with 1 moderate and 3 supporting according to ACMG guidelines, *KCNH2* has been reported as a definite or strong disease association in SQTS,^14^ and we supposed that *KCNH2*-F619V was the cause of SQTS in family 1.

Patients with SQTS in family 2 had 2 variants, *KCNH2*-H70Y and *SLC4A3*-N353K. *SLC4A3*-N353K is classified as a likely pathogenic variant. *KCNH2*-H70Y is a VUS with 1 benign strong and 2 supporting. From the report of functional analysis,^11^ *SLC4A3*-N353K, not *KCNH2*-H70Y, was considered the cause of SQTS in family 2.

### Clinical manifestations

Ventricular arrhythmia and SCD in patients with SQTS occur across a wide age range.^2^ In our study, fatal events occurred at an older age (60s) in family 1 than in family 2 (20s–30s). Events occurred during work hours in family 1, in contrast, during nighttime sleep in family 2. Notably, 2 families previously reported by Thorsen et al^15^ carrying the first identified *SLC4A3* variant also exhibited SCD during sleep at similar ages. Although SQTS lacks specific triggers, unlike long QT syndrome,^2^ the age at onset and circumstances may vary depending on the genotype.

### Characteristics of ECGs

The ECGs of these high-risk patients with SQTS demonstrated shortened QT intervals and shortened JTp intervals in both families. Although QT prolongation correlates with the risk of long QT syndrome,^16^ previous studies have indicated that the degree of QT shortening should not be used for risk stratification in these patients.^17^ Shortening of the JTp interval has been reported as a potentially diagnostic marker for SQTS.^4^ In the present study, almost all patients with SQTS exhibited markedly shortened JTp intervals, consistent with previous reports in symptomatic cohorts.^4^ However, because JTp shortening was observed uniformly across patients regardless of clinical outcome, it seems to function primarily as a diagnostic feature rather than a prognostic marker for ventricular arrhythmic events in these cases. PQD was observed in 75% of these patients and can serve as a diagnostic parameter. A previous study suggested that patients with SQTS with ER may be at a high risk of ventricular arrhythmia and SCD.^5^ This finding was also observed in our patient with SQTS who experienced SCD. Further study on SQTS is required to evaluate these factors and establish more definitive risk-stratification criteria.

The TpTec interval and TpTe/QTc ratio corrected were shorter in family 1 (*KCNH2*-F619V) than in family 2 (*SLC4A3*-N353K). Previously reported SQT1 cases also show T-wave peaks in the later portion of the T wave, similar to the T-wave morphology observed in patients with SQTS from family 1.^10^ The T-wave morphology in patients with *SLC4A3*-N353K resembled that of the reported SQT8 (*SLC4A3*-R370H, R1016G), displaying an isosceles triangular pattern.^15^^,^^18^ Thus, T-wave morphology may be useful for predicting the genotype and prognosis.

### Medical treatment for SQTS

We prescribed bepridil to the patients in family 1 with *KCNH2*-F619V. Bepridil is a multichannel blocker, and nifekalant is a selective rapid component of the delayed rectifier potassium current blocker. Both agents were associated with QT interval prolongation in our patients, consistent with a previous report.^10^ Furthermore, several antiarrhythmic drugs were associated with QT interval prolongation toward or into the normal range in patients with *SLC4A3*-N353K variants. Nifekalant suppressed VF storms, preventing the recurrence of VF in patient II-2 of family 2. We anticipate that further experimental research will advance the prevention of ventricular arrhythmia in patients with SQTS harboring *SLC4A3* variants.

We need to establish a pharmacologic management strategy and a risk-stratification model to determine implantable cardioverter-defibrillator implantation based on the results of genetic testing.

### Limitations

This observational clinical study of families with SQTS collected data during hospital visits but was limited by a small sample size, which weakens the strength of our results. Future studies with larger sample sizes and multicenter enrollments are needed.

## Conclusion

This study highlights the challenges in diagnosing and managing SQTS in patients with this rare but potentially fatal condition. Novel variants in *KCNH2* and *SLC4A3* may influence clinical features and ECG characteristics. In addition, antiarrhythmic drugs with potassium channel–blocking effects may be effective in patients with these genotypes.

## Disclosures

The authors have no conflicts of interest to disclose.

## Footnotes

Supplementary data associated with this article can be found in the online version at https://doi.org/10.1016/j.hrcr.2026.01.015.
