# Supplementary material for: Novel KCNH2 and SLC4A3 variants in short QT syndrome: Clinical phenotypes and antiarrhythmic drug response
Source: HeartRhythm Case Rep. 2026 Jan 28;12(4):464–71. doi: 10.1016/j.hrcr.2026.01.015 (PMC13100622; doi:10.1016/j.hrcr.2026.01.015)
Supplement: Supplementary Table Legend [file mmc4.docx]

**Supplementary Table 1. Clinical courses of both families 1 and 2 with SQTS**

**Supplementary Table 2.　Clinical characteristics of both families 1 and 2 with SQTS**

**Supplementary Table 3.** **ECG measurements of patients with SQTS in two families**
